# Analysis of Blood Pressure Status and Influencing Factors Among 7218 Emergency Department Nurses: An Observational Cross-Sectional Study

**DOI:** 10.1155/ijhy/4794147

**Published:** 2025-08-27

**Authors:** Wenjia Liu, Jie Liu, Jing Li, Ni Chen, Suzhi Zhang, Yufang Zhu, Yaping Wang, Xiaolin Zhang, XiaoRan Hao, Luqi Zhang, Yun Li, Bin Zhao

**Affiliations:** ^1^Office of Academic Research, The Second Hospital of Hebei Medical University, Shijiazhuang 050000, China; ^2^Department of Nursing, The Second Hospital of Hebei Medical University, Shijiazhuang 050000, China; ^3^Institute of Nursing, Hebei Medical University, Shijiazhuang 050000, China; ^4^Department of Basic Nursing, Hebei Medical University, Shijiazhuang 050000, China; ^5^Department of Epidemiology and Health Statistics, Hebei Medical University, Shijiazhuang 050000, China; ^6^Department of Nursing, Stomatological Hospital of Hebei Medical University, Shijiazhuang 050000, China; ^7^Department of Nursing, The First Hospital of Hebei Medical University, Shijiazhuang 050000, China; ^8^Department of Radiology, The Second Hospital of Hebei Medical University, Shijiazhuang 050000, China

**Keywords:** emergency department nurses, Hebei Province, hypertension, influencing factors, interactions, prevalence

## Abstract

**Background:** As frontline healthcare workers, emergency department nurses face high levels of urgency in their work and are exposed to a high risk of contingencies. Their blood pressure status and influencing factors require close attention.

**Methods:** This study employed a census method and conducted a cross-sectional survey in 11 cities in Hebei Province from November 2016 to July 2018, measuring blood pressure and collecting questionnaires on influencing factors. Binary logistic regression and multiple linear regression were used to analyze the factors influencing blood pressure. Multivariate analysis of variance was used to examine the interaction effects between monthly night shift frequency and other influencing factors on the blood pressure of emergency department nurses.

**Results:** A total of 7218 emergency department nurses in Hebei Province were included (median [IQR] age, 29 [8] years; 6038 [83.65%] women). The prevalence of hypertension was 9.43%. The median SBP (*M* [IQR]) was 112.0 (13) mmHg, and the median DBP was 70.0 (14) mmHg. Analysis showed that gender, age, BMI, marital status, hospital location, monthly night shift frequency, hyperlipidemia, and antihypertensive medication were influencing factors for the blood pressure (*p* < 0.05). Significant interactions existed between monthly night shift frequency and marital status, monthly night shift frequency and hospital grade, and monthly night shift frequency and hyperlipidemia (*p* < 0.05). The systolic blood pressure of emergency department nurses who were divorced or widowed or in Class I hospitals or hyperlipidemia increased to a high degree with the increase of night shifts. The diastolic blood pressure of those combined with hyperlipidemia increased higher with the rise of night shifts.

**Conclusion:** The blood pressure of emergency department nurses requires attention. Nursing managers should pay particular attention to nurses in the emergency department who are prone to hypertension and take proactive measures to prevent and manage hypertension.

## 1. Background

The emergency department serves as the initial point of care for critically ill patients and a central hub in the regional emergency response network. Its efficient operation directly impacts the healthcare system's emergency response capabilities. Emergency department nurses, as the frontline of this critical process, are responsible for pre-hospital emergency care, emergency observation, and resuscitation. They must promptly identify and accurately assess patients' complex conditions, rapidly initiate and efficiently execute emergency resuscitation measures, and secure the critical time window for subsequent diagnostic and therapeutic interventions [1]. However, high patient volumes and shortages of emergency department nurses have led to increased workloads, intense work rhythms, and extended working hours [2]. According to surveys, the average number of beds in the emergency departments of tertiary hospitals in China is 67, with daily patient volumes reaching up to 750, resulting in workloads for emergency department nurses that can be 2∼3 times higher than those of nurses in general wards [3]. Related studies indicate that these factors can lead to sympathetic nervous system excitation and increased catecholamine secretion, inevitably affecting blood pressure [4].

However, there is limited information on blood pressure among emergency department nurses, despite one study reporting that hypertension is relatively common among 606 Brazilian emergency department nurses [5]. Additionally, given the occupational specificity, the impact of work-related factors on blood pressure cannot be overlooked. Nevertheless, the factors influencing blood pressure in emergency department nurses identified in previous studies remain those recognized in guidelines, including age, race, body mass index (BMI), waist-to-hip ratio, and hyperlipidemia [6]. Night shifts, a common work pattern for emergency department nurses, have yet to be conclusively studied for their impact on blood pressure. Meanwhile, the interaction effects between the frequency of night shifts for emergency department nurses and other factors remain underreported. Given the objective existence of emergencies and nighttime patient needs, night shifts for emergency department nurses are unavoidable. Understanding the impact of night shifts is crucial for developing targeted hypertension prevention strategies.

Therefore, this study conducted a systematic and comprehensive survey of blood pressure among 7218 emergency department nurses, detailing the prevalence of hypertension and blood pressure levels among emergency department nurses, and exploring its influencing factors, including individual factors and work-related factors. Additionally, the study conducted a detailed analysis of the interaction effects between the frequency of night shifts among emergency department nurses and other blood pressure influencing factors, providing information to support scientific management, improve cardiovascular health levels, and enhance the quality and efficiency of emergency department nursing work.

## 2. Methods

### 2.1. Study Design and Population

The data for this study were obtained from a large cross-sectional survey conducted using a census method between November 2016 and July 2018. The survey covered all cities in Hebei Province, including Shijiazhuang City (including Xinji City), Baoding City (including Dingzhou City), Cangzhou City, Chengde City, Handan City, Hengshui City, Langfang City, Qinhuangdao City, Tangshan City, Xingtai City, and Zhangjiakou City, a total of 11 cities. Subsequently, a targeted analysis was conducted on the blood pressure status of emergency department nurses.

Inclusion criteria were defined as follows: ① registered nurses who were engaged in nursing work for ≥ 1 year and were currently working in the emergency department; ② age ≥ 18 years old; ③ no psychiatric diseases; and ④ informed consent and voluntary participation. Exclusion criteria were defined as follows: those on sick leave, maternity leave, and study leave.

### 2.2. Data Collection

#### 2.2.1. Hypertension Influencing Factors

A questionnaire survey method: The questionnaire included ① general information (gender, age, height, weight, educational background, marital status, hospital location, hospital grade [according to the Hospital Classification and Management Standards]); ② monthly night shift frequency; ③ medical history information (whether suffering from hyperlipidemia or diabetes mellitus); ④ family history of hypertension; ⑤ antihypertensive medication; and ⑥ daily life history (smoking, alcohol consumption, and exercise).

Hypertension-related factors were reported online through a questionnaire.

#### 2.2.2. Blood Pressure Measurement

Medical electronic sphygmomanometers certified by international standards or mercury sphygmomanometers validated were chosen. Standard-size cuffs with an airbag of 22–26 cm in length and 12 cm in width were used, and large-size airbag cuffs were used for obese individuals or those with large arm circumference (> 32 cm), with the airbag wrapped around at least 80% of the upper arm [7]. Each participant was asked to rest for at least 5 min, to be prohibited from smoking, to not drink coffee and tea for 30 min, and to empty the bladder. Participants should sit on the chairs with their bare upper arms at the same level as the heart and choose the right upper arm for the measurement [8]. Blood pressure was measured three times consecutively and repeated at 1–2 min each time. The average of the second and third readings was recorded, and the first blood pressure value was discarded. The blood pressure was measured by themselves and supervised by the head nurse to ensure consistency. Blood pressure values were also reported online.

### 2.3. Definitions

#### 2.3.1. Diagnostic Criteria for Hypertension

The diagnostic criteria for hypertension were referred to the Chinese Guidelines for the Prevention and Control of Hypertension (2018 Revision) [9]: systolic blood pressure (SBP) ≥ 140 mm·Hg (1 mmHg = 0.133 kPa) or diastolic blood pressure (DBP) ≥ 90 mmHg without the use of antihypertensive drugs. Previous history of hypertension: A history of hypertension, currently on antihypertensive medication, and a blood pressure < 140/90 mmHg should still be diagnosed as hypertension.

#### 2.3.2. Prevalence of Hypertension

The prevalence of hypertension is defined as the proportion of previous and new cases among survey respondents at a given time [10].

#### 2.3.3. Relevant Definitions

1. BMI: < 18.5 kg/m^2^ is considered underweight, 18.5∼< 24.0 kg/m^2^ is considered normal, 24.0∼< 28.0 kg/m^2^ considered overweight, and ≥ 28.0 kg/m^2^ is considered obese [11].
2. Monthly night shift frequency: A shift starts after 19:00 and ends before 09:00 the following morning [12]. The questions obtained the night shift frequency: “What were the average number of night shifts per month you had in the past half a year?” The results were divided into four groups for analysis: no night shifts, five or fewer night shifts per month, more than five but fewer than or equal to 10 night shifts per month, and more than 10 night shifts per month.
3. Hyperlipidemia, diabetes mellitus: “No” means no hyperlipidemia, diabetes mellitus.
4. Family history of hypertension: at least one parent has hypertension.
5. Smoking: “No” means no smoking in the past year.
6. Alcohol consumption: “No” means no consumption of any alcohol in the past year [13].
7. Exercise: running, walking, and other conscious activities lasting more than 30 min outside of working hours; “No” means no exercise and fitness activities in the past year [14].

### 2.4. Quality Control

Hebei Provincial Nursing Quality Control Center (NQCC) and the nursing department of each hospital were established as a hierarchical management mechanism to supervise the survey process. The survey team issued questionnaire notices to inform the methods of blood pressure measurements and precautions for questionnaire filling. In order to facilitate data collection and verification, this survey was conducted in batches according to geographical regions. The data were automatically uploaded to the network and returned to the NQCC after the respondents submitted. In case of missing and abnormal values, timely return visits and modifications were made to ensure the completeness and accuracy of the questionnaires. After data collection, all data were rechecked for completeness and logic to ensure accuracy.

### 2.5. Statistical Methods

All variables were statistically described. Random forest imputation was used to fill in missing value variables by R4.4.1. SPSS 27.0 software was used to analyze the data. The normality of the continuous variables was assessed. Variables following the skewed distribution were expressed by *M* (IQR), and comparisons were made using the rank-sum test. Categorical data were presented as frequencies and percentages, and a comparison was made using the *χ*^2^ test. Binary logistic regression was used to analyze the factors influencing hypertension. Multivariate linear regression analyzed SBP and DBP influencing factors. Multivariate analysis of variance was used to analyze the effect of the interaction between the monthly night shifts frequency and other blood pressure influencing factors. Differences were considered statistically significant at *p* < 0.05.

## 3. Results

### 3.1. Comparison of Hypertension Prevalence Among Emergency Department Nurses

The survey finally included 7218 emergency department nurses in Hebei Province, of which 6038 (83.65%) were female. The age range was 18∼64 years old, with a median age of 29 (8) years and a median BMI of 22.7 (5) kg/m^2^. The prevalence of hypertension among nurses in the emergency department of Hebei Province was 9.43% (681/7218), and the prevalence of hypertension was significantly higher in men (22.88%) than in women (6.81%). The blood pressure of nurses of different genders tended to increase with age and BMI. The prevalence of hypertension was higher in those with hyperlipidemia (39.21%), those with diabetes mellitus (46.84%), and those with smoking (21.14%) and alcohol consumption (10.46%), and the differences were statistically significant (*p* < 0.05), and a comparison of the prevalence of hypertension among emergency department nurses with different characteristics is shown in Table 1.

### 3.2. Emergency Department Nurses Hypertension Prevalence Influencing Factors

Binary logistic regression analysis was performed with hypertension as the dependent variable, variables that were significant in the univariate analysis and monthly night shift frequency as the independent variables. The results showed that men (compared with females, OR = 4.047, 95% CI: 3.152–5.198), age (compared with 18∼25 years old, > 25∼35 years old OR = 1.574, 95% CI: 1.076–2.302, > 35∼45 years old OR = 2.718, 95% CI: 1.713–4.311, > 45 years old OR = 3.506, 95% CI: 2.015–6.101), BMI (compared to 18.5∼24.0 kg/m^2^, 24.0∼< 28.0 kg/m^2^ OR = 1.736, 95% CI: 1.369–2.201; > 28.0 kg/m^2^ OR = 2.044, 95% CI: 1.495–2.795), and coexisting hyperlipidemia (OR = 2.910, 95% CI: 2.187–3.817 compared to those without) are risk factors for hypertension prevalence among emergency department nurses, with statistically significant differences (*p* < 0.05), as shown in Tables 2 and 3.

### 3.3. Comparison of SBP and DBP Among Emergency Department Nurses

The median level of SBP of all emergency department nursing staff was 112.0 (13) mmHg, and the median level of DBP was 70.0 (14) mmHg. The levels of SBP and DBP of male nurses were higher than those of females, and SBP and DBP increased with age and BMI, and the difference was statistically significant (*p* < 0.001). The level of blood pressure was different among educational background, marital status, hospital location and grade, and antihypertensive medication. SBP and DBP levels were elevated when hyperlipidemia, diabetes mellitus, or smoking and alcohol consumption were present, as shown in Table 4.

### 3.4. SBP and DBP Influencing Factors Among Emergency Department Nurses

Multiple linear regression analysis was performed with SBP and DBP as dependent variables, respectively, and variables that were significant in the univariate analysis and monthly night shift frequency as independent variables. The results showed that the risk factors for SBP were men (compared to women), age > 35 years (compared to 18∼25 years), BMI ≥ 24.0 kg/m^2^ (compared to 18.5∼24.0 kg/m^2^), hospital location in Tangshan (compared to Shijiazhuang), monthly night shift frequency > 5 times (compared to 0), and hyperlipidemia. The protective factor was BMI < 18.5 kg/m^2^. The risk factors for DBP were men, age > 45 years, BMI ≥ 24.0 kg/m^2^, marital status of divorced or widowed (compared to married), hospital location of Tangshan, and hyperlipidemia. The protective factor was BMI < 18.5 kg/m^2^. Antihypertensive medication was an influencing factor for the SBP and DBP of the emergency department nurses. The differences were all statistically significant (*p* < 0.05), as shown in Tables 5 and 6.

### 3.5. Interaction Analysis of the Effect of Monthly Night Shift Frequency on SBP and DBP of Emergency Department Nurses

The independent influencing factors of SBP and DBP are listed in Tables 5 and 6. Further analysis explored whether there is any interaction between the effects of each influencing factor. Rotating night shifts, as a common work pattern among nurses in the emergency department, can have an impact on blood pressure, so the interaction analysis was to explore the differences in the effect of the monthly night shift frequency on the blood pressure of the nurses in the grouping of different influencing factors, as shown in Table 7.

After adjusting for gender, age, BMI, educational background, hospital location, hospital grade, hyperlipidemia, diabetes mellitus, family history of hypertension, antihypertensive medication, smoking, alcohol consumption, and exercise, the multivariate analysis of variance was conducted with SBP and DBP as dependent variables and marital status, monthly night shift frequency, and their interaction term as independent variables. The results showed an interaction effect between marital status and monthly night shift frequency on SBP. Among emergency department nurses who were divorced or widowed, those with an average monthly night shift frequency of > 5∼10 times in the past 6 months had a SBP increase of 9.7 mmHg compared to those with > 0∼5 times. Similarly, there was an interaction effect between hospital grade and monthly night shift frequency on SBP among emergency department nurses. Among emergency department nurses in Class I hospitals, those with the monthly night shift frequency of > 0∼5 times and > 5∼10 times over the past six months had SBP elevated by 10.3 and 7.6 mmHg, respectively, compared to those with 0 times. Hyperlipidemia and monthly night shift frequency had an interactive effect on SBP and DBP in emergency department nurses. Among emergency department nurses with hyperlipidemia, those with the monthly night shift frequency of > 5∼10 times and > 10 times over the past 6 months had SBP increases of 5.6 and 7.5 mmHg, respectively, compared to those with 0 times. Those with > 5∼10 times had a DBP increase of 3.8 mmHg compared to those with 0 times. All these differences were statistically significant (*p* < 0.05).

## 4. Discussion

### 4.1. The Hypertension Prevalence of Emergency Department Nurses Is Higher Than the Overall Prevalence of Nurses in Hebei Province

We found that the hypertension prevalence of emergency department nurses was 9.4%, which was significantly higher than the overall prevalence of nurses in Hebei Province (6.9%) [15]. It may relate to the characteristics of the emergency department, highly gathered acute and critical patients, an instantly changeable working environment, and the heavy task of resuscitation and management. Emergency department nurses need to be able to respond rapidly, judge comprehensively, and dispose of accurately. Further, physical exhaustion and mental stress can cause elevated blood pressure [16]. High blood pressure is associated with various complications, affecting emergency department nurses' health status and work quality. Thus, the blood pressure of the emergency department nurses should attract widespread attention.

### 4.2. Blood Pressure Influencing Factors of Emergency Department Nurses in Hebei Province

The present study showed that men were risk factors for blood pressure in emergency department nurses, with a significantly higher prevalence of hypertension (22.88%) than women (6.81%) and higher levels of SBP and DBP in men than in women, which is in line with previous findings [17]. The relevant mechanisms may be that high androgen levels in men promote a chronic inflammatory state, resulting in vascular endothelial dysfunction [18, 19]; high levels of estrogen in women exert antihypertensive effects by promoting nitric oxide production and downregulating the levels of angiotensin II type 1 receptor and endothelial 1 receptor [20]. Other possible reasons include differences in chromosomal gene expression as well as complex social and economic factors determined by differences in the life experiences of men and women [21, 22]. Given that men are a risk factor for hypertension and that this study suggests a higher prevalence of hypertension among male emergency department nurses, nursing managers should pay attention to the blood pressure health status of male nurses, strengthen blood pressure monitoring and management for this vulnerable population, and advocate for lifestyle improvements.

The present study showed that age is a risk factor for the prevalence of hypertension in emergency department nurses, with a 10 mmHg increase in SBP in nurses aged > 45 years compared to 18∼25 years, similar to the findings of Gallagher et al. [16]. The mechanism may involve a decrease in arterial elasticity and an increase in arterial stiffness with age, leading to reduced sensitivity of pressure receptors and diminished responsiveness of the β-adrenergic system. This results in increased peripheral vascular resistance, thereby causing elevated blood pressure [23]. Older emergency department nurses are experienced and often occupy critical positions requiring high levels of decision-making and judgment, such as triage and patient assessment. Correct decisions can shorten patients' waiting times and ensure they receive timely and effective treatment. Therefore, for older emergency department nurses, shifts should be scheduled reasonably. Employee health examinations should be prioritized, and a healthy lifestyle should be promoted to slow the rate at which hypertension increases with age.

Compared with normal BMI, the risk of hypertension prevalence increased 1.736 times in overweight emergency department nurses and 2.044 times in obese ones, and both SBP and DBP increased with increasing BMI; the BMI of underweight emergency department nurses was negatively correlated with SBP and DBP, which is in line with the findings of Hirose et al. that the BMI of either males or females was correlated with the prevalence of hypertension [24]. The potential mechanisms by which overweight or obesity may lead to hypertension include: overweight or obesity causing insulin resistance, reduced release of nitric oxide by vascular endothelium, and activation of the renin–angiotensin–aldosterone system (RAAS), resulting in elevated blood pressure [25]; inflammation and oxidative stress in visceral adipose tissue surrounding blood vessels leading to microvascular dysfunction and microvascular endothelial damage, exacerbating insulin resistance and subsequently leading to elevated blood pressure [26, 27]. Emergency department nurses have busy work schedules with frequent night shifts. Research reports indicate that night shift workers may increase the frequency of nighttime meals to alleviate work-related stress, which could further contribute to weight gain and an increased risk of hypertension [28].

Hyperlipidemia is a common comorbidity of hypertension, characterized by abnormally elevated lipid levels. The positive feedback loop between the two further increases the risk of cardiovascular disease. The results of this study indicate that emergency department nurses with hyperlipidemia have an increased risk of hypertension compared to those without hyperlipidemia, consistent with the findings of Gu and Zhou [29]. Abnormally elevated lipid levels can cause endothelial cell dysfunction, impaired nitric oxide production, and impaired vascular dilation function; they can also lead to enhanced sympathetic nerve function, increased angiotensin II secretion, and elevated blood pressure [30]. Therefore, lipid control is crucial for nurses, and relevant managers should regularly conduct health education, focus on improving health literacy, and ensure that individuals with abnormal lipid levels maintain a balanced diet, engage in regular exercise, and take lipid-lowering medications when necessary.

The results of this study indicate that an increase in monthly night shift frequency leads to elevated SBP among emergency department nurses. Compared to nurses with 0 night shifts per month, those with night shift frequencies of > 0∼5 times per month and > 5∼10 times per month had increased SBP. Additionally, there was an interaction effect between monthly night shift frequency and marital status, hospital grade, and hyperlipidemia. Among emergency department nurses who were divorced, widowed, worked in Class I hospitals, or with hyperlipidemia, the increase in blood pressure associated with a higher frequency of night shifts was more pronounced. Previous studies have also shown that divorce or widowhood is associated with elevated blood pressure [31]. Divorced and widowed individuals have relatively lower levels of intimate relationships and are more susceptible to negative emotions [32–34]. The study further found that divorced or widowed individuals with a monthly night shift frequency of > 5 times had relatively higher blood pressure levels. Managers can promote blood pressure stability by implementing flexible shift schedules, strengthening social support and psychological counseling for nurses, or arranging low-intensity day shifts after high-intensity, high-frequency night shifts. The study found that the higher the night shift frequency in Class I hospitals, the higher the SBP among emergency department nurses. This suggests that managers at all levels should pay attention to the impact of night shift frequency on nurses' blood pressure, ensure reasonable scheduling, and improve the satisfaction of nurses' rest areas to ensure adequate rest. Additionally, when implementing measures such as hypertension screening and follow-up, collaboration with medical staff from cardiology and endocrinology departments can enhance intervention effectiveness. Departments that arrange scientifically reasonable night shifts and achieve target hypertension control rates for nurses can be certified and rewarded. The study found that emergency department nurses with hyperlipidemia experienced a greater increase in blood pressure with increasing night shift frequency compared to those without hyperlipidemia. This may be related to the disruption of the circadian rhythm in emergency department nurses due to inverted day–night rhythms and frequent exposure to light at night, which directly affects the physiological circadian rhythm of the hypothalamus, leading to disorders in insulin secretion and carbohydrate and lipid metabolism rhythms [35–37]. Some studies have found that increased consumption of solid tea and caffeine or irregular eating patterns during night shifts may also lead to elevated blood lipids [38, 39]. Therefore, emergency department nurses should maintain normal blood pressure through adequate sleep, balanced diet, and other healthy lifestyle practices. Additionally, managers may provide low-fat, high-protein snacks during night shifts to reduce intake of high-fat, high-sugar foods.

The study findings revealed that the location of the hospital in Tangshan is a risk factor for SBP and DBP among emergency department nurses, which may be attributed to differences in socioeconomic conditions and dietary habits across geographical regions [40]. Tangshan is a coastal city, and the proportion of seafood in the diet may be relatively high. Higher cholesterol and fat intake can increase blood pressure. Additionally, the use of antihypertensive medications is a factor influencing SBP and DBP, indicating that some emergency department nurses taking antihypertensive medication have poor blood pressure control. Educational initiatives should be implemented to encourage nurses to actively learn about chronic diseases, recognize the importance of blood pressure control, and engage in high-quality blood pressure management.

This study has certain limitations: The census method was used, and blood pressure measurements were taken using a single-day repeated measurement method, making it difficult to conduct three measurements on different days. However, after consulting experts, this blood pressure measurement method was found to be reliable and more representative. Blood pressure measurements were taken using a desktop blood pressure monitor or a mercury blood pressure monitor. Survey participants self-reported their height and weight, and no standardized measurements were conducted. However, this study employed a census method to conduct a comprehensive survey of nurses across multiple centers, strictly adhering to a tiered management mechanism, providing standardized training, and utilizing scientifically rigorous measurement methods. These measures maximized the accuracy and stability of the data. Therefore, the study results are relatively reliable, possess good representativeness, and have strong generalizability.

## 5. Conclusion

In summary, the prevalence of hypertension among emergency department nurses is higher than the overall prevalence among nurses. Gender, age, BMI, and hyperlipidemia are factors influencing the prevalence of hypertension among emergency department nurses; gender, age, BMI, hospital location, monthly night shift frequency, hyperlipidemia, and antihypertensive medication use are factors influencing SBP among emergency department nurses; gender, age, BMI, marital status, hospital location, hyperlipidemia, and antihypertensive medication are factors influencing DBP. Emergency department nurses who are divorced or widowed, work in Class I hospitals, and have hyperlipidemia experience a more significant increase in blood pressure with increased monthly night shift frequency. Managers should pay attention to the blood pressure status of the aforementioned high-risk groups for hypertension and implement effective management and support measures. Additionally, emergency department nurses who are at high risk for hypertension should actively take preventive and control measures to maintain their blood pressure within normal levels.

## Figures and Tables

**Table 1:** Comparison of hypertension prevalence among emergency department nurses with different characteristics (*n* = 7218).

Characteristics	Groups	Total (*n*, %)	Prevalence of hypertension	*χ* ^2^	*p*
Gender	Women	6038 (83.65)	411 (6.81)	298.497	< 0.001
	Men	1180 (16.35)	270 (22.88)		

Age (years)	18∼25	1434 (19.87)	57 (3.97)	309.654	< 0.001
	> 25∼35	4325 (59.92)	333 (7.70)		
	> 35∼45	1028 (14.24)	167 (16.25)		
	> 45	431 (5.97)	124 (28.77)		

BMI (kg/m^2^)	< 18.5	479 (6.64)	19 (3.97)	276.715	< 0.001
	18.5∼24.0	4165 (57.70)	227 (5.45)		
	24.0∼< 28.0	1938 (26.85)	303 (15.63)		
	≥ 28.0	636 (8.81)	132 (20.75)		

Educational background	Technical secondary school	401 (5.56)	70 (17.46)	32.175	< 0.001
	Junior college	3510 (48.63)	320 (9.12)		
	Undergraduate and above	3307 (45.82)	291 (8.80)		

Marital status	Married	5219 (72.31)	563 (10.79)	56.564	< 0.001
	Unmarried	1856 (25.71)	96 (5.17)		
	Divorced or widowed	143 (1.98)	22 (15.38)		

Hospital location	Shijiazhuang	1356 (18.79)	87 (6.42)	43.780	< 0.001
	Baoding	828 (11.47)	77 (9.30)		
	Cangzhou	701 (9.71)	76 (10.84)		
	Chengde	258 (3.57)	17 (7.36)		
	Handan	992 (13.74)	105 (10.58)		
	Hengshui	319 (4.42)	48 (15.05)		
	Langfang	572 (7.92)	45 (7.87)		
	Qinhuangdao	428 (5.93)	32 (7.48)		
	Tangshan	894 (12.39)	107 (11.99)		
	Xingtai	460 (6.37)	52 (11.30)		
	Zhangjiakou	410 (5.68)	33 (8.05)		

Hospital grade	Class I	158 (2.19)	14 (8.86)	2.235	0.327
	Class II	4023 (55.74)	398 (9.89)		
	Class III	3037 (42.08)	269 (8.86)		

Monthly night shift frequency	0	1082 (14.99)	102 (9.43)	0.807	0.848
	> 0∼5	918 (12.72)	80 (8.71)		
	> 5∼10	4776 (66.17)	459 (9.61)		
	> 10	442 (6.12)	40 (9.05)		

Hyperlipidemia	Yes	533 (7.38)	209 (39.21)	597.201	< 0.001
	No	6685 (92.62)	472 (7.06)		

Diabetes mellitus	Yes	79 (1.09)	37 (46.84)	130.760	< 0.001
	No	7139 (98.90)	644 (9.02)		

Family history of hypertension	Yes	3525 (48.84)	366 (10.38)	7.250	0.007
	No	3693 (51.16)	315 (8.53)		

Antihypertensive medication	Yes	250 (3.46)	250 (100.00)	2485.880	< 0.001
	No	6968 (96.54)	431 (6.19)		

Smoking	Yes	317 (4.39)	67 (21.14)	53.126	< 0.001
	No	6901 (95.61)	614 (8.90)		

Alcohol consumption	Yes	3852 (53.37)	403 (10.46)	10.203	< 0.001
	No	3366 (46.63)	278 (8.26)		

Exercise	Yes	5747 (79.62)	531 (9.24)	1.257	0.262
	No	1471 (20.38)	150 (10.20)		

**Table 2:** Assignment of variables for binary logistic regression analysis.

Variable	Assignment method
Gender	0 = “women”; 1 = “men”
Age (years)	1 = “18∼25”; 2 = “> 25–35”; 3 = “> 35∼45”; 4 = “> 45”
BMI (kg/m^2^)	1 = “18.5∼< 24.0”; 2 = “< 18.5”; 3 = “24.0∼< 28.0”; 4=“> 28.0”
Educational background	1 = “technical secondary school”; 2 = “junior college”; 3 = “undergraduate and above”
Marital status	1 = “married”; 2 = “unmarried”; 3 = “divorced or widowed”
Hospital location	1 = “Shijiazhuang”; 2 = “Baoding”; 3 = “Cangzhou”; 4 = “Chengde”; 5 = “Handan”; 6 = “Hengshui”; 7 = “Langfang”; 8 = “Qinhuangdao”; 9 = “Tangshan”; 10 = “Xingtai”; 11 = “Zhangjiakou”
Hospital grade	1 = “Class I”; 2 = “Class II”; 3 = “Class III”
Monthly night shift frequency	0 = “0”; 1 = “> 0∼5”; 2 = “> 5∼10”; 3 = “> 10”
Hyperlipidemia	0 = “no”; 1 = “yes”
Diabetes mellitus	0 = “no”; 1 = “yes”
Family history of hypertension	0 = “no”; 1 = “yes”
Antihypertensive medication	0 = “no”; 1 = “yes”
Smoking	0 = “no”; 1 = “yes”
Alcohol consumption	0 = “no”; 1 = “yes”
Exercise	0 = “no”; 1 = “yes”
Hypertension	0 = “no”; 1 = “yes”

**Table 3:** Binary logistic regression analysis of factors associated with hypertension prevalence among emergency department nurses.

Variables	*β*	SE	*χ* ^2^	OR	95% CI	*p*
Constant term	−4.514	0.525	73.861	0.011	—	< 0.001
Gender (compared to women)						
Men	1.398	0.128	120.036	4.047	3.152–5.198	< 0.001
Age (years, compared to 18∼25 years)						
> 25∼35	0.454	0.194	5.475	1.574	1.076–2.302	0.019
> 35∼45	1.000	0.235	18.032	2.718	1.713–4.311	< 0.001
> 45	1.255	0.283	19.712	3.506	2.015–6.101	< 0.001
BMI (kg/m^2^, compared to 18.5∼< 24.0 kg/m^2^)						
< 18.5	−0.028	0.281	0.010	0.972	0.561–1.686	0.921
24.0∼< 28.0	0.552	0.121	20.739	1.736	1.369–2.201	< 0.001
> 28.0	0.715	0.160	20.094	2.044	1.495–2.795	< 0.001
Hyperlipidemia (compared to no)						
Yes	1.068	0.146	53.788	2.910	2.187–3.871	< 0.001

**Table 4:** Comparison of systolic and diastolic blood pressure among emergency department nurses with different characteristics (*n* = 7218).

Characteristics	Groups	Systolic blood pressure (*M* (IQR), mmHg)	*Z*	*p*	Diastolic blood pressure (*M* (IQR), mmHg)	*Z*	*p*
Gender	Women	110 (15)	−33.204	< 0.001	70 (15)	−27.365	< 0.001
	Men	125 (10)			80 (10)		

Age (years)	18∼25	110 (14)	163.912	< 0.001	70 (14)	147.683	< 0.001
	> 25∼35	110 (14)			70 (15)		
	> 35∼45	117 (18)			72 (14)		
	> 45	120 (20)			80 (15)		

BMI (kg/m^2^)	< 18.5	110 (16)	854.265	< 0.001	70 (14)	640.434	< 0.001
	18.5∼< 24.0	110 (17)			70 (16)		
	24.0∼< 28.0	120 (20)			78 (10)		
	≥ 28.0	120 (14)			80 (15)		

Educational background	Technical secondary school	120 (19)	14.806	< 0.001	76 (10)	26.708	< 0.001
	Junior college	112 (12)			70 (14)		
	Undergraduate and above	112 (14)			70 (15)		

Marital status	Married	115 (12)	29.225	< 0.001	70 (14)	16.823	< 0.001
	Unmarried	110 (14)			70 (15)		
	Divorced or widowed	115 (16)			75 (10)		

Hospital location	Shijiazhuang	110 (15)	55.368	< 0.001	70 (15)	57.001	< 0.001
	Baoding	112 (13)			70 (15)		
	Cangzhou	115 (15)			70 (15)		
	Chengde	110 (13)			70 (12)		
	Handan	115 (15)			70 (15)		
	Hengshui	120 (15)			75 (10)		
	Langfang	110 (11)			70 (13)		
	Qinhuangdao	11 (14)			70 (12)		
	Tangshan	116 (10)			74 (10)		
	Xingtai	115 (14)			72 (12)		
	Zhangjiakou	110 (18)			70 (15)		

Hospital grade	Class I	110 (10)	37.094	< 0.001	70 (10)	2.210	0.331
	Class II	110 (14)			70 (12)		
	Class III	116 (16)			70 (15)		

Monthly night shift frequency	0	110 (15)	7.780	0.051	70 (14)	7.768	0.051
	> 0∼5	115 (14)			70 (15)		
	> 5∼10	113 (13)			70 (15)		
	> 10	115 (10)			74 (10)		

Hyperlipidemia	Yes	130 (21)	−20.172	< 0.001	80 (15)	−19.161	< 0.001
	No	110 (14)			70 (15)		

Diabetes mellitus	Yes	121 (20)	−6.846	< 0.001	80 (18)	−7.007	< 0.001
	No	112 (13)			70 (14)		

Family history of hypertension	Yes	113 (13)	−1.287	0.198	70 (14)	−0.775	0.438
	No	112 (13)			70 (14)		

Antihypertensive medication	Yes	140 (15)	−25.059	< 0.001	90 (10)	−24.503	< 0.001
	No	110 (14)			70 (15)		

Smoking	Yes	120 (20)	−10.632	< 0.001	80 (15)	−9.487	< 0.001
	No	111 (14)			70 (15)		

Alcohol consumption	Yes	115 (14)	−3.700	< 0.001	70 (13)	−3.943	< 0.001
	No	110 (14)			70 (15)		

Exercise	Yes	113 (13)	−0.122	0.903	70 (15)	−1.240	0.215
	No	112 (12)			70 (13)		

**Table 5:** Results of multiple linear regression analysis of systolic blood pressure influencing factors among emergency department nurses.

Variables	*B*	SE	*β*	*t*	*p*
Constant term	107.558	1.203		89.390	< 0.001
Gender (compared to women)					
Men	10.587	0.392	0.289	27.026	< 0.001
Age (years, compared to 18∼25 years)					
> 35∼45	1.396	0.554	0.036	2.519	0.012
> 45	4.649	0.724	0.081	6.423	< 0.001
BMI (kg/m^2^, compared to 18.5∼< 24.0 kg/m^2^)					
< 18.5	−2.380	0.532	−0.044	−4.470	< 0.001
24.0∼< 28.0	4.620	0.312	0.151	14.809	< 0.001
≥ 28.0	7.167	0.481	0.150	14.884	< 0.001
Hospital location (compared to Shijiazhuang)					
Tangshan	1.458	0.480	0.036	2.519	0.012
Monthly night shift frequency (compared to 0)					
> 5∼10	0.995	0.384	0.035	2.591	0.010
> 10	1.503	0.638	0.027	2.356	0.019
Hyperlipidemia (compared to no)	4.704	0.537	0.091	8.752	< 0.001
Antihypertensive medication (compared to no)	22.730	0.756	0.307	30.081	< 0.001

*Note:R* ^2^ = 0.353, adjusted *R*^2^ = 0.350, *F* = 118.921, *p* < 0.001.

**Table 6:** Results of multiple linear regression analysis of diastolic blood pressure–influencing factors among emergency department nurses.

Variables	*B*	SE	*β*	*t*	*p*
Constant term	70.083	0.940		74.565	< 0.001
Gender (compared to women)					
Men	6.573	0.287	0.242	22.908	< 0.001
Age (years, compared to 18–25 years)					
> 45	2.475	0.565	0.058	4.380	< 0.001
BMI (kg/m^2^, compared to 18.5∼< 24.0 kg/m^2^)					
< 18.5	−1.716	0.416	−0.043	−4.129	< 0.001
24.0∼< 28.0	2.913	0.244	0.129	11.961	< 0.001
≥ 28.0	4.645	0.376	0.131	12.361	< 0.001
Marital status (compared to married)					
Divorced or widowed	1.968	0.726	0.027	2.710	0.007
Hospital location (compared to Shijiazhuang)					
Tangshan	1.311	0.375	0.043	3.493	< 0.001
Hyperlipidemia (compared to no)	3.471	0.420	0.090	8.269	< 0.001
Antihypertensive medication (compared to no)	16.181	0.590	0.295	27.414	< 0.001

*Note:R* ^2^ = 0.283, adjusted *R*^2^ = 0.279, *F* = 85.830, *p* < 0.001.

**Table 7:** Interaction analysis of monthly night shift frequency and other blood pressure–influencing factors on systolic and diastolic blood pressure of emergency department nurses.

Variables		Systolic blood pressure (mmHg)				Diastolic blood pressure (mmHg)			
		Average value	Standard error	*F*	*p*	Average value	Standard error	*F*	*p*
Marital status	Monthly night shift frequency			2.156	0.044			1.675	0.123
Married	0	132.1	0.874	2.524	0.056	85.9	0.683	1.288	0.277
	> 0∼5	132.9	0.909			86.7	0.710		
	> 5∼10	133.3	0.815			86.2	0.637		
	> 10	133.9	1.021			86.2	0.798		
Unmarried				1.039	0.374			1.194	0.310
	0	131.8	1.269			86.9	0.992		
	> 0∼5	132.9	1.078			87.0	0.842		
	> 5∼10	133.5	0.867			86.5	0.677		
	> 10	133.2	1.200			87.9	0.937		
Divorced or widowed				3.516	0.014			2.027	0.108
	0	133.7	2.698			88.5	2.108		
	> 0∼5	126.4	2.930			83.3	2.289		
	> 5∼10	136.1^a^	1.364			89.0	1.065		
	> 10	135.6	3.553			86.9	2.776		
Hospital grade				3.259	0.003			1.055	0.388
Class I				4.604	0.003			1.866	0.133
	0	126.7	2.156			84.3	1.686		
	> 0∼5	137.0^b^	2.939			87.4	2.298		
	> 5∼10	134.3^c^	1.337			88.4	1.046		
	> 10	135.1	3.941			87.2	3.082		
Class II				1.809	0.143			1.177	0.317
	0	132.6	0.917			86.9	0.717		
	> 0∼5	133.9	0.952			87.6	0.744		
	> 5∼10	133.0	0.832			86.9	0.650		
	> 10	133.7	0.984			87.5	0.770		
Class III				3.064	0.027			0.268	0.849
	0	133.4	0.978			86.1	0.765		
	> 0∼5	133.5	0.972			86.6	0.760		
	> 5∼10	134.8	0.840			86.5	0.657		
	> 10	135.2	1.401			86.4	1.095		
Hyperlipidemia				5.540	< 0.001			4.995	0.002
Yes				7.446	< 0.001			1.176	0.317
	0	131.5	1.353			85.9	1.057		
	> 0∼5	135.1	1.613			88.6	1.260		
	> 5∼10	137.1^d^	0.981			89.7	0.767^f^		
	> 10	139.0^e^	2.014			88.1	1.573		
No				1.029	0.379			4.949	0.002
	0	130.7	0.952			85.3	0.744		
	> 0∼5	131.4	0.960			85.7	0.750		
	> 5∼10	131.3	0.894			85.2	0.699		< 0.001
	> 10	131.7	1.032			85.8	0.806		

^a^Statistically significant difference in systolic blood pressure of emergency department nurses in the divorced or widowed group with a frequency of > 5∼10 night shifts compared with > 0∼5 times.

^b^Statistically significant difference in systolic blood pressure of emergency department nurses in Class I hospital with a frequency of > 0∼5 night shifts compared with 0 times.

^c^Statistically significant difference in systolic blood pressure of emergency department nurses in Class I hospital with a frequency of > 5∼10 night shifts compared with 0 times.

^d^Statistically significant difference in systolic blood pressure of emergency department nurses in the hyperlipidemia group with a frequency of > 5∼10 night shifts compared with 0 times.

^e^Statistically significant difference in systolic blood pressure of emergency department nurses in the hyperlipidemia group with a frequency of > 10 night shifts compared with 0 times.

^f^Statistically significant difference in diastolic blood pressure of emergency department nurses in the hyperlipidemia group with a frequency of > 5∼10 night shifts compared with 0 times.

## Data Availability

The data that support the findings of this study are available upon request from the corresponding author. The data are not publicly available due to privacy or ethical restrictions.

## Nomenclature

SBP: Systolic blood pressure
DBP: Diastolic blood pressure
OR: Odds ratio
NQCC: Nursing Quality Control Center
IQR: Interquartile range
